# Metabolic syndrome components are associated with oxidative stress in overweight and obese patients

**DOI:** 10.20945/2359-3997000000036

**Published:** 2018-05-07

**Authors:** Nayara Rampazzo Morelli, Bruna Miglioranza Scavuzzi, Lucia Helena da Silva Miglioranza, Marcell Alysson Batisti Lozovoy, Andréa Name Colado Simão, Isaias Dichi

**Affiliations:** 1 Universidade Estadual de Londrina Universidade Estadual de Londrina Departamento de Pós-Graduação em Ciências da Saúde Londrina PR Brasil Departamento de Pós-Graduação em Ciências da Saúde, Universidade Estadual de Londrina (UEL), Londrina, PR, Brasil; 2 Universidade Estadual de Londrina Universidade Estadual de Londrina Departamento de Ciência e Tecnologia de Alimentos Londrina PR Brasil Departamento de Ciência e Tecnologia de Alimentos, Universidade Estadual de Londrina (UEL), Londrina, PR, Brasil; 3 Universidade Estadual de Londrina Universidade Estadual de Londrina Departamento de Patologia, Análises Clínicas e Toxicológicas Londrina PR Brasil Departamento de Patologia, Análises Clínicas e Toxicológicas, Universidade Estadual de Londrina (UEL), Londrina, PR, Brasil; 4 Universidade Estadual de Londrina Universidade Estadual de Londrina Departamento de Medicina Interna Londrina PR Brasil Departamento de Medicina Interna, Universidade Estadual de Londrina (UEL), Londrina, PR, Brasil

**Keywords:** Overweight, obesity, metabolic syndrome, oxidative stress, nitrosative stress

## Abstract

**Objective:**

The aim of this study is to evaluate the influence of the body mass index (BMI) and the metabolic syndrome (MetS) parameters on oxidative and nitrosative stress in overweight and obese subjects.

**Subjects and methods:**

Individuals were divided into three groups: the control group (G1, n = 131) with a BMI between 20 and 24.9 kg/m^2^, the overweight group (G2, n = 120) with a BMI between 25 and 29.9 kg/m^2^ and the obese group (G3, n = 79) with a BMI ≥ 30 kg/m^2^.

**Results:**

G3 presented higher advanced oxidation protein products (AOPPs) in relation to G1 and G2 (p = 0.001 and p = 0.011, respectively) whereas G2 and G3 had lower levels of nitric oxide (NO) (p = 0.009 and p = 0.048, respectively) compared to G1. Adjusted for the presence of MetS to evaluate its influence, the levels of AOPPs did not differ between the groups, whereas NO remained significantly lower. Data adjusted by the BMI showed that subjects with higher triacylglycerol levels had higher AOPPs (p = 0.001) and decreased total radical-trapping antioxidant parameter/uric Acid (p = 0.036). Subjects with lower high-density lipoprotein (HDL) levels and patients with higher blood pressure showed increased AOPPs (p = 0.001 and p = 0.034, respectively) and lower NO levels (p = 0.017 and p = 0.043, respectively). Subjects who presented insulin resistance had higher AOPPs (p = 0.024).

**Conclusions:**

Nitrosative stress was related to BMI, and protein oxidation and nitrosative stress were related to metabolic changes and hypertension. MetS components were essential participants in oxidative and nitrosative stress in overweight and obese subjects.

## INTRODUCTION

Obesity and overweight are chronic disorders of multifactorial origin, which can be defined as an increase in the accumulation of body fat (1). Changes in lifestyle and diet have resulted in an increased number of overweight and obese subjects in developed and developing countries (2). This trend has been verified in practically all ages, genders and ethnicities (2). Therefore, overweight and obesity have emerged as two of the largest public health problems worldwide. Excess body weight is associated with an increased risk of developing metabolic syndrome (MetS). MetS is a complex disorder that is represented by a cluster of cardiovascular risk factors that are associated with central fat deposition, abnormal plasma lipid levels, elevated blood pressure, insulin resistance and a low-grade inflammatory state. MetS has also been associated with increased oxidative and nitrosative stress (3).

The harmful effects of free radicals, which are mainly represented by reactive oxygen species (ROS) or reactive nitrogen species, have been implicated in the physiopathology of overweight, obesity, hypertension, endothelial dysfunction, and MetS (4,5), suggesting that oxidative stress can be the underlying mechanism of this dysfunctional metabolic picture in obese subjects (6). In addition, high ROS production and the decrease in antioxidant capacity leads to various abnormalities. These abnormalities include endothelial dysfunction – which is characterized by a reduction in the bioavailability of vasodilators, particularly nitric oxide (NO) (7), and an increase in endothelium-derived contractile factors, favoring atherosclerotic disease (1).

Although markers of oxidative stress have been studied in obese and overweight patients with and without MetS, we are not aware to date of studies evaluating the influence of body weight on oxo-nitrosative stress in the other components of MetS.

In a previous study performed by our group, we verified that an increase in oxidative stress is mostly attributable to obesity in patients with MetS (8), but this is not the case in overweight subjects without MetS. It is therefore unclear whether metabolic changes of obesity, referred to as metabolic obesity, are independent risk factors for increased oxo-nitrosative stress than the other components of MetS. In order to extend the data of the mentioned study, the objective of the present study was to evaluate the influence of the body mass index (BMI) on oxidative and nitrosative stress in overweight and obese subjects and to verify whether the presence of the components of MetS would modify the results.

## SUBJECTS AND MATERIALS

### Subjects

Patients from the Internal Medicine Ambulatory of the University Hospital of Londrina, Paraná, Brazil were chosen to participate in this cross-sectional study. Three hundred and thirty patients agreed to participate in the study. Inclusion criteria were patients (both genders) aged from 18 to 65 years. Exclusion criteria were thyroid, renal, hepatic, gastrointestinal, infectious or oncological diseases and the use of lipid-lowering drugs, drugs for hyperglycemia, anti-inflammatory drugs, hormone replacement therapy, and antioxidant supplements. For ethical reasons, patients who were taking antihypertensive drugs were not excluded and were allowed to continue taking the same dose of the drugs.

The patients were divided into three groups: the control group included 131 subjects with a BMI between 20 and 24.9 kg/m^2^. The overweight group consisted of 120 subjects with a BMI between 25 and 29.9 kg/m^2^, and the group with obesity consisted of 79 subjects with a BMI ≥ 30. MetS was defined following the Adult Treatment Panel III criteria. A diagnosis of MetS was arrived at for subjects with at least three of the following five characteristics: (1) abdominal obesity, which was defined as a waist circumference (WC) ≥ 102 cm in men and ≥ 88 cm in women; (2) hypertriglyceridemia, which was defined as triglycerides ≥ 150 mg/dL; (3) low levels of high-density lipoprotein (HDL) cholesterol, which was defined as HDL ≤ 40 mg/dL in men and ≤ 50 mg/dL in women; (4) high blood pressure, which was defined as blood pressure ≥ 130/85 mmHg; and (5) high-fasting glucose, which was defined as glucose ≥ 100 mg/dL.

### Ethics, consent, health and safety

The research was conducted in an ethical and responsible manner and is in full compliance with all relevant codes of experimentation. In addition, the Ethical Committee of the University of Londrina – Paraná, Brazil – approved all procedures involving human participants (185/2013). This clinical investigation was conducted according to the principles expressed in the Declaration of Helsinki.

Written informed consent was obtained from all the participants, who acknowledged that they cannot be identified via the paper and that they are fully anonymized.

All mandatory laboratory health and safety procedures have been complied.

### Anthropometric and blood pressure measurements

Anthropometric measurements and laboratorial parameters were assessed. Body weight was measured to the nearest 0.1 kg in the morning through the use of an electronic scale, with individuals wearing light clothing and no shoes; height was measured to the nearest 0.1 cm through the use of a stadiometer. BMI was calculated as weight (kg) divided by height (m) squared. WC was measured on standing subjects midway between the lowest rib and the iliac crest. Three blood pressure measurements taken with a 1-min interval after the participant had been seated were recorded on the left arm. The mean of these measurements was used in the analysis. We considered the current use of antihypertensive medication as an indication of high blood pressure.

### Biochemical, immunological, and hematological biomarkers

After fasting for 12 hours, the subjects underwent the following laboratory blood analysis evaluated through a biochemical auto-analyzer (Dimension Dade AR Dade Behring, Deerfield, IL, USA) using Dade Behring^®^ kits: total cholesterol, HDL, low-density lipoprotein (LDL), triacylglycerol (TG), glucose and uric acid (UA). Plasma insulin level was determined by chemiluminescence microparticule immunoassay (Architect, Abbott Laboratory, Abbott Park, IL, USA). The homeostasis model assessment insulin resistance (HOMA-IR) was used as a surrogate measurement of insulin sensitivity. HOMA-IR = fasting insulin (U/ml) x fasting glucose (mmol/L)/22.5. IR was considered when HOMA-IR ≥ 2.5 (9).

### Oxidative and nitrosative stress measurements

Samples for evaluating oxidative stress and total antioxidant capacity were analyzed with e thylenediamine tetraacetic acid as an anticoagulant and antioxidant. All samples were centrifuged at 3,000 rpm for 15 minutes, and plasma aliquots were stored at −70ºC until assayed. All stress measurements were performed in triplicate.

### Tert-butyl hydroperoxide-initiated chemiluminescence (CL-LOOH)

CL-LOOH in plasma was evaluated as described previously by Gonzales Flecha and cols. (10). CL-LOOH is considered to be much more sensitive and specific than the thiobarbituric acid reactive substances method, the usual method to determine lipid oxidation. For the CL measurement, reaction mixtures were placed in 20-mL scintillation vials (low-potassium glass) containing final concentrations of plasma (250 uL), 30 mM KH2PO4/K2HPO4 buffer (pH 7.4), and 120 mM KCl with 3 mM of LOOH in a final volume of 2 mL. CL-LOOH was measured in a Beckman LS 6000 liquid scintillation counter set to the out-of-coincidence mode, with a response of 300 to 620 nm. The vials were kept in the dark until the moment of assay, and determination was carried out in a dark room at 30°C. The results were expressed in counts per minute.

### Determination of advanced oxidation protein products (AOPPs)

AOPPs were determined in the plasma using the semi-automated method described by Witko-Sarsat and cols. (11). AOPP concentrations were expressed as micromoles per liter (µmol/L) of chloramines-T equivalents.

### Total radical-trapping antioxidant parameter (TRAP)

TRAP was determined as reported by Repetto and cols. (12). This method detects hydrosoluble and liposoluble plasma antioxidants by measuring the chemiluminescence inhibition time induced by 2,2-azobis (2-amidinopropane). The system was calibrated with the vitamin E analog TROLOX, and the values of TRAP were expressed in the equivalent of μM Trolox/mg UA. TRAP measurements in conditions associated with hyperuricemia, such as MetS, may be inaccurate because the UA concentration accounts for 60% of the total plasma antioxidant capacity. Some reports have verified an unexpected increase in TRAP in MetS subjects (13). Thus, a correction of TRAP based on UA concentration was performed (13).

### Determination of sulfhydryl (SH) groups of proteins

SH groups of proteins were evaluated in plasma samples through a spectrophotometric assay based on 2,2-dithiobisnitrobenzoic acid (DTNB), as reported previously (14), and the results are expressed in μM.

### Evaluation of nitric oxide metabolites (NOx)

The NO concentration in a sample was estimated by measuring the NOx in nitrites (NO2^-^) and nitrates (NO3^-^) using cadmium beads for the reduction of nitrate to nitrite. The concentrations of these metabolites were later determined according to the method proposed by Griess (15). The values were expressed in µM.

### Statistical analysis

Categorical data were analyzed through a chi-squared test or, when appropriate, through Fisher's exact test, and data were expressed in absolute values. The Kolmogorov-Smirnov test was used to assess the normality of distribution. All continuous variables presented non-parametric distribution, even after logarithmic transformation. The comparisons of the three groups categorized by BMI were performed through the use of the non-parametric Kruskal-Wallis test with the *post-hoc* Dunn test. The variables that presented significance in the univariate analysis of variance were included in the multinomial logistic regression to verify which oxidative stress parameters were associated with BMI. The Mann-Whitney test was used to compare two groups, and a logistic binary regression analysis was performed to adjust for age, sex, ethnicity and BMI. The results were considered significant when P < 0.05. A statistical analysis program, SPSS version 20.0, was used for evaluations.

## RESULTS

**Figure 1:**
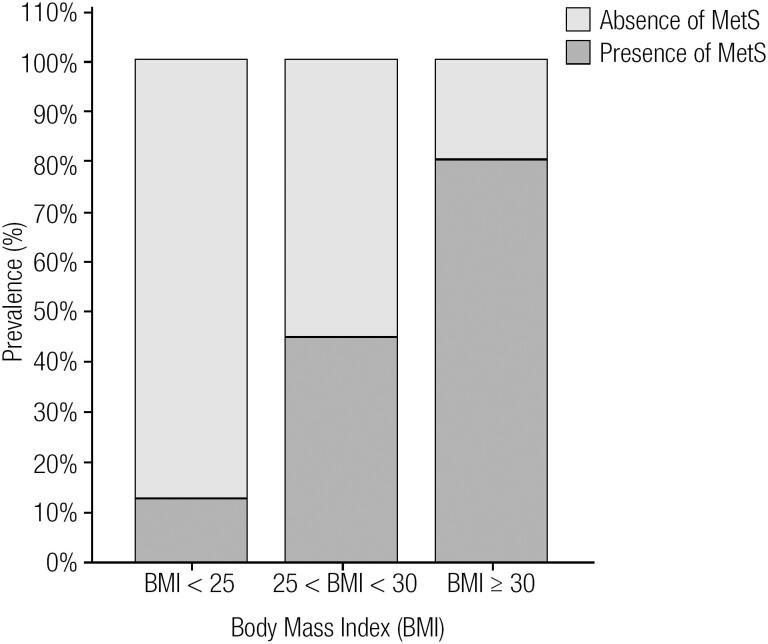
Prevalence of the metabolic syndrome across body mass index categories. Normal weight, < 25 kg/m^2^; overweight, 25-30 kg/m^2^; and obese, ≥ 30 kg/m^2^.

There was no statistically significant difference in ethnicity between the three groups (Table 1). Overweight (G2) and obese subjects (G3) did not differ regarding sex and age. However, the control group (G1) had a higher frequency of women compared to the subjects in G2 (p < 0.0001) and G3 (p < 0.05), and they were younger (p < 0.0001) than those in the G2 and G3 groups. The presence of MetS was higher (p < 0.0001) in G3 compared to G1 and G2 and in G2 compared to G1 (Table 1 and Figure 1). G3 presented higher WC (p < 0.0001, p < 0.0001), glucose (p < 0.0001, p < 0.05), insulin (p < 0.001, p < 0.001), HOMA-IR (p < 0.001, p < 0.001), and TG (p < 0.001, p < 0.01) and decreased HDL–cholesterol (p < 0.001, p < 0.05) levels compared to G1 and G2, respectively (Table 1). Meanwhile, G2 had higher WC (p < 0.0001), glucose (p < 0.001), insulin, HOMA-IR, and TG and decreased (p < 0.001) HDL-cholesterol levels compared to G1. G2 and G3 showed higher total cholesterol (p < 0.05) and LDL cholesterol (p < 0.01) levels compared to those of G1 (Table 1).

**Table 1:** Clinical and laboratory characteristics of controls (G1), overweight (G2) and obese subjects (G3)

	G1 (n = 131)	G2 (n = 120)	G3 (n = 79)	G1 X G2	G1 X G3	G2 X G3
Gender (% men)	18	43	32	**< 0.0001**	**< 0.05**	0.1231
Ethnicity (% caucasians)	80	78	82	0.8420	0.8525	0.6180
MetS (Y/N)	12/119	65/55	72/7	**< 0.0001**	**< 0.0001**	**< 0.0001**
Age	32.0 (25.0-43.0)	43.0 (34.5-53.0)	43.0 (34.0-50.0)	**< 0.001**	**< 0.001**	NS
BMI (kg/m^2^)	22.04 (20.90-23.57)	27.12 (25.98-28.33)	32.25 (31.04-34.99)	**<0.001**	**< 0.001**	**< 0.001**
WC (cm)	82.0 (77.0-88.0)	97.0 (91.0-101.0)	108.0 (103.0-115.0)	**< 0.0001**	**< 0.0001**	**< 0.0001**
Fating glucose (mg/dL)	86.0 (83.0-92.0)	92.0 (85.0-98.0)	98.0 (88.0-107.0)	**< 0.001**	**< 0.001**	**< 0.05**
Insulin (U/mL)	6.4 (4.65-8.80)	8.10 (5.90-12.90)	14.5 (10.9-17.4)	**< 0.001**	**< 0.001**	**< 0.001**
HOMA-IR	1.338 (1,020-1,940)	1.989 (1.285-3.238)	3.234 (2.696-4.781)	**< 0.001**	**< 0.001**	**< 0.001**
Total cholesterol (mg/dL)	186.0 (152.5-209,0)	197.0 (168.0-226.0)	198.0 (180.0-224.0)	**< 0.050**	**< 0.010**	NS
HDL-cholesterol (mg/dL)	56.5 (48.5-67.0)	46.5 (38.0-59.5)	42.0 (37.0-51.0)	**< 0.001**	**< 0.001**	**< 0.05**
LDL-cholesterol (mg/dL)	109.8 (83.8-130.2)	121.0 (95.2-141.1)	125.3 (100.5-142.0)	**< 0.050**	**< 0.010**	NS
Triacylglycerol (mg/dL)	74.5 (48.5-107.5)	124.0 (90.0-183.0)	175.0 (127.0-231.0)	**< 0.001**	**< 0.001**	**< 0.01**

MetS: metabolic syndrome; BMI: body mass index; WC: waist circumference; IR: insulin resistance; HOMA: homeostasis model assessment; HDL: high-density lipoprotein; LDL: low-density lipoprotein; NS: nonsignificant; WC: waist circumference.

Table 2 shows the results of oxidative stress in the three studied groups with p values adjusted for sex and age. G3 presented higher AOPP values in relation to G1 and G2 (p = 0.001 and p = 0.011, respectively), whereas significantly lower NOx values were found in G2 and G3 when compared to those of G1 (p = 0.009 and p = 0.048, respectively). The groups were then adjusted for the presence of MetS to evaluate its influence on the results. In this new analysis, AOPPs did not differ between the groups, whereas significant lower NO maintained its significance. Lipid hydroperoxides and TRAP/UA did not have any significant change in the groups. Table 3 shows the results obtained after performing multiple regressive stepwise analyses to clarify the importance of body weight on oxidative and nitrosative stress; AOPPs were not significant after adjusting for sex, age and the presence of MetS when comparing controls and overweight, controls and obese and overweight and obese (0.777; 0.750; 0.483, respectively). NO maintained its significance when comparing controls and overweight subjects (0.007; 0.064; 0.799, respectively).

**Table 2:** Oxidative stress evaluation in controls (G1), overweight (G2) and obese subjects (G3)

	G1 (n = 131)	G2 (n = 120)	G3 (n = 79)	G1 X G2*	G1 X G3*	G2 X G3*
Hydroperoxides (cpm)	13900 (10740-17010)	14120 (10950-17350)	13540 (10250-16260)	NS	NS	NS
AOPP (µmol/L)	127.2 (98.2-174.4)	159.5 (124.7-230.3)	195.2 (157.3.257.1)	NS	0.001	0.011
NO (μM)	25.67 (13.67-40.45)	13.83 (8.17-42.84)	12.10 (7.96-27.63)	0.009	0.048	NS
TRAP/UA (μM Trolox/mg/dL)	177.1 (147.2-207.5)	158.8 (126.9-190.0)	138.0 (115.3-164.9)	NS	NS	NS

AOPP: advanced oxidation protein products; NO: nitric oxide; TRAP: total radical-trapping antioxidant parameter; UA: uric acid; NS: nonsignificant.

* Adjusted p value for sex and age. AOPP was not significant after adjusting for the presence of MetS, whereas NO maintained its significance.

**Table 3:** Stepway analysis for oxidative stress evaluation in controls (G1), overweight (G2) and obese subjects (G3) BMI

Parameters		Wald	p*	Odds-Ratio	Confidence interval
Gender	G1 x G2 G1 x G3 G2 x G3	14.294 0.944 6.174	**< 0.0001** 0.331 **0.013**	3.635 1.501 2.422	1.862 – 7.098 0.662 – 3.404 1.205 – 4.868
Age	G1 x G2 G1 x G3 G2 x G3	20.998 5.46 3.211	**< 0.0001** **0.019** 0.073	1.067 1.040 1.026	1.038 – 1.097 1.006 – 1.074 0.998 – 1.056
MetS	G1 x G2 G1 x G3 G2 x G3	13.538 53.367 21.823	**< 0.0001** **< 0.0001** **< 0.0001**	4.308 29.759 0.145	1.979 – 9.378 11.974 – 73.956 0.064 – 0.326
AOPP	G1 x G2 G1 x G3 G2 x G3	0.080 0.102 0.491	0.777 0.750 0.483	0.999 1.001 0.999	0.995 – 1.004 0.996 – 1.005 0.995 – 1.002
NO	G1 x G2 G1 x G3 G2 x G3	7.377 3.440 0.065	**0.007** 0.064 0.799	1.018 1.016 1.002	1.005 – 1.031 0.99 – 1.032 0.987 – 1.017

MetS: metabolic syndrome; AOPP: advanced oxidation protein products; NO: nitric oxide.

* Adjusted p value for sex, age and presence of MetS.

AOPP was not significant after adjusting for sex and age, whereas NO maintained its significance when comparing controls and overweight subjects.

To verify the association between oxidative stress biomarkers and the presence of MetS, a binary logistic regression was performed and was adjusted for sex and age. The AOPP levels were directly associated with the presence of MetS (Wald = 16.039, df = 1, OR = 1.009, 95% CI = 1.005-1.009, p < 0.0001), and the NO values were inversely associated (Wald = 18.941, df = 1, OR = 0.958, 95% CI = 0.940-0.977, p < 0.0001) with the presence of MetS (data not shown).

The association between the oxidative stress parameters and the individual components of MetS was measured, and the values were adjusted by BMI, sex, age and ethnicity; the results are shown in Table 4. Subjects with higher TG levels had higher AOPPs (p = 0.001) and decreased TRAP/UA levels (p = 0.036) compared to individuals without hypertriacylglycerolemia. Subjects with lower HDL cholesterol and patients with higher blood pressure levels showed increased AOPPs (p = 0.001 and p = 0.034, respectively) and lower NO levels (p = 0.017 and p = 0.043, respectively) compared to individuals without low HDL-cholesterol levels and with normal blood pressure. Subjects who presented insulin resistance had higher AOPP levels (p = 0.024) compared to those without insulin resistance.

**Table 4:** Oxidative stress evaluation according to the components of the metabolic syndrome

	Gender (%men)	Ethnicity (% cauc)	Age	BMI (Kg/m^2^)	LOOH (cpm)	AOPP (µmol/L)	NO (μM)	TRAP/UA (μM Trolox/mg/dL)
TG < 150 mg/dL n = 218	22	81	36.0 (28.0-47.0)	24.42 (21.74-27.68)	14120 (10900-17730)	134.2 (101.2-174.7)	23.72 (12.29-42.84)	170.7 (138.4-204.1)
TG ≥ 150 mg/dL n = 108	46	78	46.0 (37.0-53.0)	29.75 (26.63-32.10)	13570 (8549-18750)	225.1 (160.1-275-89)	11.19 (6.60-27.63)	137.8 (118.0-173.0)
p	< 0.001	0.6321	< 0.0001	< 0.0001	NS	< 0.0001	< 0.0001	< 0.0001
* Adjusted p	----	----	----	----	NS	**0.001**	NS	**0.036**
Normal HDL n = 186	26	80	38.5 (30.0-47.0)	24.36 (21.91-28.04)	14290 (10800-18110)	136.8 (102.9-181.2)	25.78 (12.38-43.93)	163.9 (138.0-205.1)
Reduced HDL n = 137	35	80	42.0 (30.5-50.0)	27.99 (25.44-31.60)	13650 (11480-16720)	183.8 (131.4-256.5)	12.37 (7.53-29.91)	149.6 (122.4-186.3)
p	NS	NS	NS	< 0.0001	NS	< 0.0001	< 0.0001	0.0011
* Adjusted p	----	----	----	----	----	**0.001**	**0.017**	NS
Normotensive n = 217	27	80	35.0 (27.0-44.0)	24.80 (21.83-28.04)	13750 (11130-17650)	134.4 (100.4-183.8)	22.84 (11.90-40.23)	166.3 (137.8-200.9)
Hypertensive n = 112	36	79	47.0 (39.0-55.0)	28.60 (26.17-31.70)	14550 (10010-18190)	195.7 (154.3-274.2)	11.95 (6.80-33.73)	145.7 (119.5-184.8)
p	0.1414	0.9926	< 0.0001	< 0.0001	0.7971	< 0.0001	0.0010	0.0010
* Adjusted p	----	----	----	----	NS	**0.034**	**0.043**	NS
Without IR n = 163	7	23	38 (29.0-47.0)	24.22 (21.76-26.67)	14200 (11070-17890)	137.4 (104.0-184.9)	23.5 (11.91-41.00)	169.6 (138.0-203.2)
With IR n = 115	36	22	42 (30.0-53.0)	30.05 (27.21-32.92)	13550 (9291-16690)	182 (127.9-256.5)	11.78 (7.07-27.74)	146.3 (116.3-179.6)
p	0.0781	NS	0.023	< 0.0001	NS	< 0.0001	< 0.0001	< 0.0001
* Adjusted p	----	----	----	----	----	**0.024**	NS	NS

Cauc: Caucasian; BMI: body mass index; IR: insulin resistance; LOOH: hydroperoxides; AOPP: advanced oxidation protein products; NO: nitric oxide; TRAP: total radical-trapping antioxidant parameter; UA: uric acid; TG: tryacylglycerols; NS: nonsignificant.

* Binary logistic regression adjusted for sex, age, ethnicity and BMI.

Multiple regressive stepwise analyses were performed (Table 5). After adjusting for sex, age and the presence of MetS, AOPPs maintained their significance for TG, HDL, blood pressure and HOMA-IR (< 0.0001, < 0.0001, 0.017, and 0.024, respectively); NO maintained its significance for HDL (0.025), and TRAP maintained its significance for TG (0.034).

**Table 5:** Multiple regressive stepwise analysis for oxidative stress evaluation in different components of the metabolic syndrome

	Parameters	Wald	p	Odds-Ratio	Confidence interval
TG	Sex	8.613	**0.003**	0.289	0.126 – 0.662
	Age	0.098	0.755	1.005	0.972 – 1.040
	BMI	21.695	< 0.0001	1.212	1.118 – 1.314
	AOPP	20.380	< 0.0001	1.011	1.006 – 1.016
	NO	1.082	0.298	0.992	0.977 – 1.007
	SH	0.104	0.748	1.949	0.033 – 113.506
	TRAP-UA	4.495	**0.034**	0.990	0.980 – 0.999
HDL	BMI	13.434	**< 0.0001**	1.113	1.051 – 1.179
	AOPP	14.431	**< 0.0001**	1.006	1.003 – 1.010
	NO	5.006	**0.025**	0.987	0.975 – 0.998
	TRAP-UA	0.062	0.803	0.999	0.993 – 1.005
Blood Pressure	Age	20.787	**< 0.0001**	1.086	1.048 – 1.125
	BMI	16.929	**< 0.0001**	1.167	1.084 – 1.256
	AOPP	5.683	**0.017**	1.005	1.001 – 1.009
	NO	3.509	0.061	1.013	0.999 – 1.028
	SH	0.737	0.391	4.993	0.127 – 196.458
	TRAP-UA	1.586	0.208	0.995	0.987 – 1.003
HOMA-IR	Age	4.753	**0.029**	0.969	0.0942 – 0.997
	BMI	57.168	**< 0.0001**	1.463	1.325 – 1.614
	AOPP	5.092	**0.024**	1.004	1.001 – 1.008
	NO	1.574	0.210	0.991	0.977 – 1.005
	TRAP-UA	0.370	0.543	0.998	0.990 – 1.005

TG: triacylglycerol; HDL: high-density lipoprotein; HOMA-IR: homeostasis model assessment-insulin resistance; BMI: body mass index; AOPP: advanced oxidation protein products; NO: nitric oxide; Sulfhydryl (SH) groups of proteins; TRAP-UA: total radical-trapping antioxidant parameter-uric acid.

After adjusting for sex and age AOPP maintained its significance for TG, HDL, hypertension and HOMA-IR, NO maintained its significance for HDL and TRAP-AU maintained its significance for TG. Whereas the other oxidative and nitrosative stress markers lost significance.

## DISCUSSION

The redox state was similar in healthy, overweight and obese subjects when controlled for the presence of MetS, and therefore, the principal finding of the present study was that oxidative stress evaluated through lipid and protein oxidation in patients with obesity is mainly related to the presence of MetS and is less related to BMI. However, nitrosative stress with decreased NO bioavailability was associated with BMI, independently of the presence of MetS. In addition, this study verified that protein oxidation was associated with several individual components of MetS, including insulin resistance.

The present data are partially in agreement with our previous study, which showed that increases in oxidative stress markers in overweight subjects were only verified in the presence of MetS (8). However, in that study, obese subjects and nitrosative stress were not evaluated, which differs from the conditions of the present study. Thus, the impact of this original work lays in the observation that only nitrosative stress was related to BMI, whereas protein oxidation was related to each component of MetS.

Although an increase in oxidative stress in patients with obesity is an undisputed issue and can be caused by several factors (16), the present study is in line with others, which pointed out the utmost importance of the presence of MetS to reinforce this association. Skalicky and cols. (17) verified in obese subjects and Krzystek-Korpacka and cols. (18) verified in overweight and obese adolescents that oxidative stress seemed to be increased through a combination of risk factors associated with MetS rather than by obesity per se. Fujita and cols. (19) demonstrated that values of oxidative stress increased with the number of components of MetS. Taken together, these data suggest that – although weight gain or visceral fat may contribute, to some extent, to an increase in oxidative stress – the presence of MetS is fundamental in showing ROS augmentation in overweight and obese subjects.

Our data are also in accordance with that of previous studies, which showed that hypertriacylglycerolemia, hypertension, lower HDL cholesterol values and insulin resistance are essential factors in provoking oxidative stress (13,19,20). Obesity and IR are considered key factors for the development of MetS. There is mounting evidence that oxidative stress is involved in the development of insulin resistance and that, once IR is acquired, all the other components of MetS could be developed as a result (21,22). Hypertriacylglycerolemia and hypertension lead to an increased production of superoxide anion (O_2_^-^) via the nicotinamide adenosine diphosphate oxidase pathway. This anion reacts rapidly with NO to form peroxynitrite (ONOO^-^) – thus inactivating NO and leading to endothelial dysfunction, one of the mechanisms responsible for hypertension in these patients (23) – whereas HDL cholesterol antioxidant activity, a major mechanism mediating its cardioprotective effect, is impaired (20). Of note, in the current study, hypertriacylglycerolemia showed the highest degree of redox imbalance, as it was the only MetS component, and it concomitantly increased protein oxidation and decreased antioxidant capacity.

Our results show an association between the presence of insulin resistance and increased levels of protein oxidation. Although several reports have established the importance of insulin resistance and oxidative stress in the development of both diabetes and cardiovascular disease (24,25), the precise role of oxidative stress as a cause or consequence of insulin resistance is still debated. Furukawa and cols. (26) demonstrated in cultured adipocytes that elevated levels of fatty acids increased oxidative stress via NADPH oxidase activation, and oxidative stress caused dysregulated production of adipocytokines – including adiponectin, plasminogen activator inhibitor-1, IL-6, and monocyte chemotactic protein-1. In addition, in mice with obesity, treatment with an NADPH oxidase inhibitor reduced ROS production in adipose tissue; attenuated the dysregulation of adipocytokines; and improved diabetes, hyperlipidemia, and hepatic steatosis. NADPH oxidase inhibitors could improve insulin sensitivity via the suppression of the effects induced through chronic exposure to ROS. These results suggested that increased oxidative stress in accumulated fat is an early instigator of MetS and that the redox state in adipose tissue is a potentially useful therapeutic target for obesity-associated MetS. In addition, hydrogen peroxide impairs insulin signaling and inhibits glucose transport, two cardinal features of insulin resistance (27). On the other hand, insulin itself promotes hydrogen peroxide formation in human fat cells (4). Altogether, it is tempting to speculate that oxidative stress can be both cause and consequence of insulin resistance (16,28).

It has been suggested that AOPPs are an early marker of MetS and are the most appropriate parameter for the determination of oxidative stress in MetS patients (29). AOPPs are formed during oxidative stress through the action of chlorinated oxidants, mainly hypochlorous acid and chloramines, produced by myeloperoxidase in activated neutrophils (11). AOPPs are structurally similar to advanced glycation end products (AGEs) and exert similar biological activities to those of AGEs – i.e., induction of pro-inflammatory cytokines and adhesion molecules (11). The present study showing that AOPPs were associated with metabolic changes and hypertension is in line with the importance of protein oxidation in patients with these components of MetS, independent of BMI, and confirm our previous finding that protein oxidation is more related to MetS parameters than lipid oxidation (30). Of note, BMI does not participate in the definition of the MetS and does not have a robust association verified between WC and insulin resistance. Although some studies have reported an association between AOPPs and BMI (13,30), the current study only presented this finding when the groups were not adjusted for the presence of MetS.

Changes in NOx levels have been linked with disorders of metabolic and cardiovascular homeostasis that can culminate in obesity-induced IR and endothelial dysfunction. Serum NOx were shown to be increased in MetS and be associated with other metabolic components such as BP, BMI, waist-to-hip ratio, and fasting plasma glucose in cluster analyses (31). In addition to overproduced NO, reduced levels of NO may also be a risk factor for the development of cardiometabolic disease (32). Although endothelial dysfunction has been considered an important issue in patients with obesity, the results of studies on NOx levels have been contradictory. Whereas some reports have shown higher NO levels (33), others have found the opposite results, similarly to the present study (34). NO is synthesized in endothelial cells by endothelial nitric oxide synthase (eNOS) activity, and it is responsible for vasodilatation and the maintenance of endothelial function; eNOS is expressed constitutively and synthesizes NO in only small amounts under basal conditions. In contrast, oxidative stress provokes inducible nitric oxide synthase (iNOS) expression even in low-grade inflammatory conditions, such as obesity, and consequently increases NO – which would be consumed in a reaction with superoxide anion, yielding peroxynitrite (35). This hypothesis is supported by some authors who demonstrated an increase in nitrotyrosine, a marker of endogenous peroxinitrite generation (36). Thus, the balance between eNOS and iNOS could explain NO increases or decreases in obese subjects. Although oxidative stress may induce NO production, the NO decrease associated with BMI found in the present study is probably related to higher NO consumption through oxidative stress, reducing NO bioavailability. Furthermore, previous investigations have shown that HDL induces a variety of signaling events – involving scavenger receptor B type I, cholesterol efflux and the stimulation of the phosphorylation of eNOS – that lead to the activation and increased expression of eNOS and the subsequent production of NO. Thus, the decreased NO levels in the present study may be related to the HDL cholesterol reduction (37,38).

Overweight is highly associated with arterial hypertension, independently from the occurrence of MetS, and a BMI of 25 kg/m^2^ or greater accounted for approximately 34% and 62% of hypertension in men and in women, respectively (35). NO plays a major role in regulating blood pressure, and its deficient bioactivity is an important component of hypertension (37). Hypertensive subjects have increased generation of ROS – which scavenge NO, thereby reducing NO bioavailability (23). This study confirms the well-established relationship between NO decreases and hypertension, independent of whether BMI is considered.

The following limitations have to be considered in the present study. The first limitation is the small number of participants. Second, the food pattern and physical activities of the individuals were not measured. Third, the antihypertensive drugs the patients were taking, such as angiotensin-converting enzyme inhibitors, may elevate plasma adiponectin levels, which in turn can increase NO levels (23). Fourth, additional tests to evaluate oxidative and, especially, nitrosative stress would make our data more consistent. Nevertheless, the present study also has several strengths. First, to our knowledge, this is the first study to evaluate concomitantly oxidative and nitrosative stress in overweight and obese subjects. Second, we adjusted the results of oxidative stress measurements for the presence of MetS to evaluate its influence on the results.

In conclusion, only nitrosative stress was related to BMI, whereas protein oxidation was related to each component of MetS. In addition, both NO and advanced oxidative protein products were related to hypertension. In general, MetS components were essential participants in overweight and obese subjects, but hypertriacylglycerolemia was the parameter that showed the highest degree of redox imbalance. Although more studies are warranted to confirm the present data, this study reinforces the importance of concomitantly analyzing oxidative and nitrosative stress to obtain a more complete picture of overweight, obesity and associated conditions.
